# HEALTHY LEISURE: ADVANCING UNDERSTANDING OF LEISURE DECISIONS, ACTIVITIES, AND HEALTH CONSEQUENCES

**DOI:** 10.1093/geroni/igac059.050

**Published:** 2022-12-20

**Authors:** Claire Smith, Soomi Lee, Allison Bielak

## Abstract

Leisure activities promote healthy aging, yet aging poses new challenges to engagement in active, meaningful, health-promoting leisure. This stalemate wherein people need to but struggle to engage in healthy leisure as they age calls for further research describing what healthy leisure entails, decision processes predicting it, and long-term consequences over the lifespan. This symposium brings together five rigorous studies aimed at understanding diverse aspects of leisure – how adults make daily leisure decisions, the health-promoting or demoting consequences of specific leisure activities as a person ages, and unique challenges faced by vulnerable populations. Paper 1 describes how daily energy and affect conjointly influence the types and variety of leisure activities midlife adults choose. Paper 2 identifies specific leisure activities that protect against cognitive decline in older adults, with consideration of vulnerable subgroups (illiterate, rural residents). Paper 3 investigates how seven leisure activities longitudinally predict cognitive impairment in older adults and differences by life course SES. Paper 4 identifies social interactions with friends as a key aspect of healthy leisure that longitudinally associates with cognitive function in older adults. Paper 5 focuses on a vulnerable population, older breast cancer survivors, and examines whether participating in cognitively stimulating leisure activities is associated with cognitive function (vs. non-cancer controls). These papers use different samples and datasets to describe the nature, causes, and consequences healthy leisure by specific population groups. The discussant, Dr. Allison Bielak will integrate key findings from these studies, discuss their theoretical and methodological contributions, and consider opportunities for future research.

